# Glycosylation as a design component of biomaterials in implant design

**DOI:** 10.1016/j.mmr.2026.100070

**Published:** 2026-09-29

**Authors:** Abhay Pandit

**Affiliations:** Rinn Medical Devices, University of Galway, Galway H91 W2TY, Ireland

**Keywords:** Glycosylation, Glycan, Biomaterial, Medical device, Host response, Implant design, Patient stratification, Sialylation

## Abstract

An implanted medical device is specified in detail through its bulk and surface chemistry, mechanical properties, architecture, degradation kinetics, and surface ligands, whereas the host receiving it is specified hardly at all. Much of what the host presents at that interface is glycan, and glycan-mediated recognition acts through defined receptor families, including the Siglecs, the C-type lectin receptors, the galectins, and the regulators of complement. The host glycome is not fixed. It is assembled combinatorially, varies with age, metabolic state, and inflammation, differs between recipients, and is altered systematically in disease. Those alterations are measurable before implantation and are resolved both regionally and temporally, *N*-glycosylation differing region by region within the human Parkinsonian nigrostriatum and *α*-2,6-sialylation rising from 19.5% of the *N*-glycan pool in healthy intervertebral disc to 23%−24% in degenerated tissue. This Perspective argues that glycosylation should be specified as a component of the biomaterials from which devices are built. Because a diseased glycome is typically altered in several respects at once, a design need not address all of it, and a single motif may be selected where it is quantitatively dominant, mechanistically implicated, and chemically tractable. Three propositions follow: host glycophenotype is a measurable covariate of outcome and a basis for patient stratification; glycan structure, sulfation, and presentation density are specifiable material properties, with sulfation tunable independently of backbone; the analytical methods required to control a glycan attribute are established, so that the obstacle is convention rather than capability.

An implanted device meets the body through its biomaterial surface, and that biomaterial is specified by convention in a fixed order beginning with bulk and surface chemistry and proceeding through mechanical properties, architecture, and degradation kinetics to the surface ligands. The order has served device design for four decades. It describes the device closely and the host barely at all, although the majority of proteins an implant meets, recruits, or provokes the host to secrete are glycoproteins whose glycan structures are almost never specified, controlled or reported.

## Glycan recognition at the device interface

Dense and heterogeneous glycan display is a universal feature of cellular surfaces, and glycan-mediated recognition governs a wide range of post-implantation events [1]. Four receptor systems are relevant. Terminal sialic acids engage the Siglecs, which bear immunoreceptor tyrosine-based inhibitory motifs and deliver restraining signals to macrophages, neutrophils, and natural killer cells. Mannosylated and fucosylated structures are recognised by C-type lectin receptors on antigen-presenting cells. Sialylated surfaces recruit complement factor H and restrain alternative pathway activation, a principal mechanism of self-discrimination. Galectins cross-link glycoconjugates into lattices that govern receptor residency and have been proposed as targets for immune-engineered biomaterials [2].

These systems bear directly on fibrotic encapsulation, the commonest mode of functional failure for implanted devices, since the macrophage phenotypes determining it are regulated through lectin engagement [2]. A material presenting defined glycans therefore acts on the receptors that determine whether an implant is encapsulated or integrated, rather than on stiffness or topography as indirect proxies.

## The host glycome is a variable, not a constant

Unlike the polypeptide, the glycan is not template-encoded but assembled combinatorially by competing glycosyltransferases and glycosidases whose expression varies with metabolic state, age, hypoxia, and inflammation. Glycosylation is altered systematically across a wide range of disease states in patterns specific enough to serve as diagnostic markers [3], and the glycophenotype of injured and inflamed tissue is now sufficiently characterised to be treated as a property of the disease [4]. The relevant alterations are those of the tissue and cell surfaces contacted by the device, not those of the circulating proteome, so the defining specimen is tissue from the disease state rather than serum.

Those alterations are resolved regionally and temporally. Mapping the human Parkinsonian nigrostriatal system, we found *N*-glycosylation altered region by region, each region carrying a distinct signature with associated transcriptomic and proteomic changes [5]; in rodent brain, spatial *N*-glycan distribution shifts measurably upon striatal neuroinflammation [6]. In the human intervertebral disc, hydrophilic interaction chromatography of released *N*-glycans resolved 283 glycan species across 48 peaks in healthy (Pfirrmann grade I) and degenerated (grade IV/V) tissues, *n*=6 per group, with a further 77 *N*-glycans resolved spatially by matrix-assisted laser desorption ionisation imaging, extending an earlier finding that nucleus pulposus cells of the degenerated human intervertebral disc carry altered sialylated and fucosylated motifs associated with inflammation and degeneration [7]. Degeneration shifted the pool from oligomannose and tetraantennary species toward mono- and biantennary sialylated structures, and *α*-2,6-sialylation rose from 19.57% and 19.46% in healthy annulus fibrosus and nucleus pulposus to 24.05% and 23.10% in the degenerated equivalents [8].

The structural detail is informative for design. Structures follow Oxford notation, in which F denotes a core fucose, A the number of antennae, G the number of galactose residues, S the number of sialic acids, GalNAc an *N*-acetylgalactosamine residue, and Man an oligomannose glycan carrying the stated number of mannose residues. In degenerated nucleus pulposus, the oligomannose species Man5 and Man8, and the triantennary disialylated FA3G3S2, were significantly reduced, whereas the biantennary FA2G2 and FA2F1G1GalNAc1, and the monosialylated FA2G2S1, were increased, with outer-arm fucosylation also trending upward; lectin histochemistry agreed, *Sambucus nigra* agglutinin reporting *α*-2,6-sialylation and *Ulex europaeus* agglutinin *α*-1,2-fucose. Transcriptionally, the shift tracked upregulation, under cytokine stimulation, of the sialyltransferase genes *ST3GAL1* and *ST3GAL4* (ST3 beta-galactoside alpha-2,3-sialyltransferase 1 and 4) and *ST6GALNAC4* (ST6 *N*-acetylgalactosaminide alpha-2,6-sialyltransferase 4), and of the fucosyltransferase gene *FUT11* (fucosyltransferase 11) [8]. A design targeting this tissue is therefore not choosing among undifferentiated sugars but among identified structures with identified biosynthetic routes.

## Selecting a single target from a heterogeneous glycophenotype

Heterogeneity of this kind does not oblige a design to address all of it. Differential glycan expression in diseased tissue is typically multiple, but a strategy need not match that complexity. Three criteria identify a workable target: 1) it should be quantitatively dominant within the altered pool; 2) it should be mechanistically implicated in the pathology rather than correlated with it; and 3) it should be tractable to a chemistry that can be carried on a material. Abundance alone is insufficient, since the most conspicuous alteration in a diseased tissue need not be the one driving the pathology.

Hypersialylation in the degenerated disc satisfies all three. It is quantitatively dominant in the figures above, and its mechanistic contribution has been tested directly. Inhibition of sialylation in human nucleus pulposus cells *in vitro* with 3Fax-peracetyl Neu5Ac altered 1575 genes in degenerated cells and 2881 in cytokine-stimulated healthy cells, with significant reduction of tumour necrosis factor-α signalling, inflammatory response, and epithelial-to-mesenchymal transition pathways [8]. The same tissue has been addressed from the materials side, a hyaluronic acid hydrogel having prevented the pain phenotype in a rat model of disc injury [9]. The diseased glycome is therefore a point of intervention as well as a description, and glyco-modulatory chemistry can be incorporated into the biomaterial rather than delivered separately.

## Glycans as specifiable properties of the biomaterial

Table 1 [1], [3], [5], [7], [8], [10], [11], [12], [13], [14] maps particular glycan modifications onto biomaterial classes and device application scenarios. Two parameters are specifiable. The first is sulfation, which is carried on the polymer backbone and is tunable independently of backbone identity. Alginate sulfates immobilised at degrees of substitution from 0 to 2.7 bound fibroblast growth factor 2 (FGF-2) in proportion to substitution as measured by quartz crystal microbalance with dissipation monitoring, and the highest degree of substitution gave the greatest proliferation of A172, SH-SY5Y, and PC-12 cells and the greatest neurite outgrowth (*P*<0.05 for all lines) [10]. Tuned instead for chemokine affinity, a star polyethylene glycol (PEG)-heparin network captured monocyte chemoattractant protein 1 and interleukin-8 from chronic venous ulcer fluid and improved healing in diabetic mice beyond the approved comparator Promogran [11].

**Table 1:** Glycan modifications classified by biomaterial class and device application scenario.

**Glycan modification**	**Biomaterial class**	**Device application scenario**	**Reported effect**	**Reference**
**Host glycophenotype as the design target**				
Region-specific *N*-glycome of diseased tissue	Injectable and scaffold-based CNS delivery	Neural implants; Neurodegenerative disease sites	*N*-glycosylation differs region by region in the human Parkinsonian nigrostriatum; Regional signatures differ within one organ	[5]
Sialylated and fucosylated *N*-glycan motifs	Disc repair scaffolds and injectables	Degenerated intervertebral disc	Altered motifs in human nucleus pulposus cells implicated in inflammation and degeneration	[7]
Disease-associated tissue glycosylation generally	Any host-contacting biomaterial	Recipient stratification at enrolment	Alterations specific enough to be diagnostic; Supports glycophenotype as an outcome covariate	[3]
**Glycan presented by the biomaterial**				
Sulfation, degree of substitution 0−2.7	Alginate sulfate coatings (non-heparin backbone)	Growth-factor-retaining and neural interface surfaces	FGF-2 binding rises with degree of sulfation; Highest degree of substitution gives greatest proliferation and neurite outgrowth	[10]
Sulfation tuned for chemokine affinity	StarPEG-heparin hydrogel network	Chronic wound contact layer	Sequesters inflammatory chemokines from human wound exudate; Healing restored beyond an approved comparator	[11]
Terminal sialic acid (Siglec ligand)	Surface-functionalised implant coatings	Immunomodulation at any implant bed	Inhibitory signalling to macrophages, neutrophils and natural killer cells	[1]
Mannose and fucose (C-type lectin ligand)	Particulate and scaffold surfaces	Antigen-presenting cell targeting; Immune-directed devices	Recognition by C-type lectin receptors on antigen-presenting cells	[1]
Sialylation density (complement regulation)	Blood-contacting surfaces	Vascular grafts, catheters, oxygenators	Factor H recruitment restrains alternative pathway activation	[1]
Multivalent display: valency and ligand spacing	Synthetic glycocalyx-engineered surfaces	Cell-instructive surfaces; Biosensing substrates	Monovalent affinity weak (μmol/L−mmol/L); Avidity and selectivity set by valency, spacing, and scaffold conformation	[12]
Batch glycosignature of the raw material	Commercial pepsin-soluble type I collagen	Any collagen-based scaffold, film, gel, or coating	Batches share mannose- and galactose-reactive signals but differ markedly in fucosylated and sialylated epitopes; Lectin profiling gives accessible quality control	[13]
**Glyco-modulation as an active device function**				
*α*-2,6-sialylation (inhibited)	Small-molecule glyco-modulator for incorporation into delivery systems	Intervertebral disc regeneration	Hypersialylation marks the degenerated disc; Inhibition lowers oxidative stress and inflammatory signalling in nucleus pulposus cells	[8]
Core fucose removed (Fc *N*-glycan, Asn297)	Biologic component of drug-device combinations	Combination products; Local biologic delivery	FcγRIIIa affinity and antibody-dependent cellular cytotoxicity raised by orders of magnitude; Approved products exist	[14]

CNS. Central nervous system; Fc. Fragment crystallisable; FcγRIIIa. Fc gamma receptor IIIa; FGF-2. Fibroblast growth factor 2; PEG. Polyethylene glycol.

The second parameter is presentation. Individual glycan-lectin interactions are weak, typically micromolar to millimolar, and selectivity is generated by multivalency, so avidity depends on valency, ligand spacing, and the conformational freedom of the presenting surface [12]. A glycan grafted at arbitrary density therefore specifies little. Matrices designed against these parameters now constitute a defined class, in that collagen hydrogels functionalised with defined glycans alter the glycoenvironment of primary neuronal culture and change cell behaviour accordingly [15], while glyco-modulatory matrix-inspired materials have been reviewed as a category in their own right [16].

Synthetic glycocalyx engineering extends the same logic, permitting glycan structures to be presented at a spatial precision that conventional molecular approaches cannot achieve, and allowing valency and spacing to be varied independently of the underlying chemistry [12]. The practical consequence is that two materials of identical composition may differ in biological effect solely through the arrangement of their pendant sugars, which is a specification parameter rather than a manufacturing incidental.

## Knowing the substrate

Two stages precede specification. The first stage concerns the raw material. Materials of natural origin carry intrinsic biological function, and identifying it, retaining it through processing and verifying its survival is distinct from characterising bulk properties [17]. The glycosignature of common raw materials is frequently unknown. Profiling three production batches of commercial pepsin-soluble type I collagen with a panel of fifteen lectins, combined with enzyme-linked lectin sorbent assay and lectin-probed Western blotting, we found batches sharing mannose- and galactose-reactive signals but differing markedly in low-abundance fucosylated and sialylated epitopes; specificity was confirmed by haptenic sugar inhibition and by digestion with *α*-1,6-fucosidase and *α*-2,3-neuraminidase, and the complex signals arose most likely from co-purified matrix glycoproteins rather than the collagen α-chains [13]. This variability is not detected by the release tests ordinarily applied to collagen.

Functional consequences were partly separable from the analytical ones. All collagen-coated surfaces strongly promoted MDA-MB-231 adhesion irrespective of batch, and glycosidase treatment produced batch-dependent trends in wound-closure kinetics that did not reach significance at matched time points [13]. The analytical heterogeneity is therefore established more firmly than its functional consequence, which is the honest position and defines the experiment still required.

The second stage concerns the target, defined empirically in human tissue from the relevant disease state rather than inferred from healthy controls or animal models.

## Glycophenotype as a stratifying variable

If the response to a device is transduced through lectin and complement receptors reading host glycans, glycophenotype is a covariate of outcome and can be measured at enrolment rather than inferred retrospectively from subgroup analysis. Implementation requires a pre-specified covariate, a defined assay and threshold, and stratified randomisation or stratified analysis, as is routine in trials stratifying on a molecular marker. The phenotype is not static, since inflammation strongly modifies glycosylation [3], so the landscape at implantation differs from that at one year; a material whose glyco-modulatory function is weighted toward the inflammatory phase and reverts thereafter follows from that observation.

The assay defining such a covariate must sample the correct compartment. Circulating immunoglobulin G glycosylation is measurable at scale and responds to systemic inflammation, but it reports the plasma cell compartment rather than the cell surfaces within the implant bed, and the two need not move together. The specimen defining a device-relevant target is therefore tissue taken from the disease state and analysed by methods preserving spatial information wherever the anatomy is heterogeneous, as both the nigrostriatal and the disc series required [5], [8]. A covariate usable at enrolment must also be obtainable at enrolment, which restricts the approach in the first instance to procedures in which diseased tissue is excised or biopsied as part of the intervention, such as discectomy, arthroplasty or surgical debridement, and in which that tissue is at present discarded. The same specimens would supply the distribution of the marker across the target population, without which a threshold cannot be pre-specified.

## Toward a controlled glycan attribute

Precedent exists in the biopharmaceutical sector. Removal of core fucose from the fragment crystallisable (Fc) *N*-glycan at Asn297 increases affinity for Fc gamma receptor IIIa (FcγRIIIa) and antibody-dependent cellular cytotoxicity by orders of magnitude, and glycoengineered antibodies built on this principle hold regulatory approval [14]. Glycosylation is there a critical quality attribute, controlled in manufacture, released against specification and audited. Five steps would transfer the discipline to devices: 1) a defined glycan attribute enters the design input alongside modulus and degradation profile; 2) an analytical method of demonstrated specificity and reproducibility supports it; 3) incoming raw material is released against a glycosignature rather than protein content alone; 4) process validation demonstrates that manufacturing does not destroy the attribute; and 5) glycomic characterisation of retrieved devices and peri-implant tissue confirms whether the attribute behaved as intended.

The analytical requirement is less forbidding than often assumed. Glycans cannot be amplified and are not template-derived, and a single site may carry dozens of structures differing only in linkage or monosaccharide position, so full characterisation requires released-glycan chromatography with mass spectrometric detection, exoglycosidase digestion to resolve linkage and, where spatial information is required, imaging mass spectrometry. These methods are established, and the spatial variant resolved the regional signatures above [5], [6], [8]. For routine control, a lectin panel is sufficient to expose batch variability undetected by conventional release testing [13].

## Limitations

Three limitations qualify the argument. First, most of the human glycomic evidence is cross-sectional and associative, establishing that glycosylation differs between diseased and healthy tissue without establishing direction of causation, and the one interventional dataset available here is *in vitro* [8]. Second, the sample sizes in tissue glycomics remain small, 6 per group in the disc series, reflecting the difficulty of obtaining graded human tissue rather than any methodological limit. Third, no prospective study has yet recorded recipient glycophenotype against device outcome, so the covariate proposed here is argued from mechanism rather than demonstrated from trial data. Each of these is addressable with existing methods, and the third is the one which would settle the question.

## Conclusions

Three changes in practice follow. The diseased glycome, resolved regionally and defined in human tissue, should enter the device design specification. Glycan structure, sulfation, and presentation density should be reported alongside modulus and degradation profile. Recipient glycophenotype should be recorded as a stratifying variable in implant trials. Much of the evidence remains associative, although targeted inhibition of a disease glycophenotype has now been shown to alter the accompanying biology [8]. What it identifies is a determinant of host response that is measurable, patient-specific and at present neither measured nor controlled. Specifying a device against the glycome of the tissue that will receive it, and directing it to recipients whose glycophenotype suits it, is achievable with methods already in routine laboratory use.

## Abbreviations

CNS. Central nervous system;

Fc. Fragment crystallisable;

FcγRIIIa. Fc gamma receptor IIIa;

FGF-2. Fibroblast growth factor 2;

PEG. Polyethylene glycol.

## Declarations

### Ethics approval and consent to participate

Not applicable.

## Funding

This publication has emanated from research conducted with the financial support of Taighde Éireann – Research Ireland, under Grant number 13/RC/2073_P2 at CÚRAM Research Ireland Centre for Medical Devices and Grant number 23/RC/12208 at Rinn Medical Devices, Ireland.

## Competing interests

The author declares that there are no competing interests.

## Data Availability

Not applicable.
